# Storage Stability of Meat Analogs Supplemented with Vegetable Oils

**DOI:** 10.3390/foods12193586

**Published:** 2023-09-27

**Authors:** Youngjae Cho, Junhwan Bae, Jiseon Lee, Mi-Jung Choi

**Affiliations:** 1 Department of Food Science and Technology, Pusan National University, Miryang 50463, Republic of Korea; moonjae@pusan.ac.kr; 2 Department of Food Science and Biotechnology of Animal Resources, Konkuk University, Seoul 05029, Republic of Korea; royal__style@naver.com; 3 Carbohydrate Bioproduct Research Center, Sejong University, Seoul 05006, Republic of Korea; mango021@naver.com

**Keywords:** vegetable analog, vegetable oil, storage stability, freeze storage

## Abstract

The addition of various oils to meat analog has been an important topic to improving its juiciness and tenderness. However, this causes a concern about oil leaching from the meat analog during long-term storage. Here, we aimed to assess the storage stability of vegetable-oil-supplemented meat analogs and analyze the effects of temperature and storage period on their physiochemical characteristics. The meat analogs were prepared by adding 30 g castor oil, orange oil, palm oil, shortening, or margarine vegetable oil based on 100 g of textured vegetable protein. They were then stored at −18 or −60 °C for 6 months and analyzed at one-month intervals. The meat analog supplemented with orange oil had the highest water content (64.85%; 66.07%), hardness (35.48 N; 34.05 N), and DPPH-radical-scavenging activity (30.01%; 30.87%) under −18 and −60 °C, respectively, as well as the highest liquid-holding capacity in different conditions. During frozen storage, temperature barely affected the meat quality. The storage stability of all meat analog samples was maintained for 6 months, although the quality was slightly reduced with an increase in storage duration. Coliform group bacteria were not detected regardless of the storage condition. In conclusion, all results supposed that orange oil can be a promising candidate for improving the juiciness and tenderness of meat analogs, and the quality of samples was maintained for at least 6 months under frozen storage. The findings of this study are relevant to the development and promotion of meat analog as an alternative to animal meat.

## 1. Introduction

Global meat production is approximately 19 billion kg per year. The per capita meat consumed in 2017 was 8.2 kg, but this is predicted to increase to 8.6 kg in 2025 [1,2]. While the demand for meat is steadily increasing, resources such as land and water for raising livestock are limited, which limits supply [3,4]. Greenhouse gases emitted during livestock production are one of the important causes of environmental problems. Methane (~37%) and nitrous oxide (N_2_O; ~65%) are the greatest contributors to global greenhouse gas emissions [5]. The steady increase in carbon dioxide (CO_2_) emissions [6] is contributing to global warming, thereby further worsening the environment. Besides the effects of meat production on the environment, meat consumption is associated with health issues. Meat is high in cholesterol and saturated fat and can cause high blood pressure, obesity, cardiovascular disease, and cancer [7,8,9].

Owing to its adverse effects on health and the environment, concerns have been raised regarding meat consumption, and the need to produce alternatives to meat has been emphasized. This has led to an increased interest in meat alternatives based on plant protein. Research on developing meat substitutes has drawn the attention of scientists. Studies are being conducted to replace meat with soy protein [10,11,12,13]. However, risks and limitations associated with the production of meat analogs also exist [14]. Among them, consumer attitudes towards substituting meat with plant-based alternatives can be the most important risk to meat analog producers. However, plant meat products are less juicy than real meat because they have much less fat, which plays a major role in juiciness [15]. Due to this reason, plant meat products need to increase the juiciness of meat analogs using different oils, particularly to improve consumer acceptability of meat analog.

Among vegetable oils, shortening and margarine have compositions similar to those of animal oils because they are artificially hydrogenated to form saturated fats [16]. Thus, shortening and margarine, with relatively high saturated fat contents, appear to be suitable candidates for use in the development of plant meat analogs. Orange oil, a highly unsaturated and volatile flavoring ingredient, can have a masking effect on the bean odor of soybean in meat substitutes. Ahmad et al. [17] reported the use of orange oil as a flavoring ingredient to weaken the odor. Owing to its higher viscosity than that of other vegetable oils [18], castor oil is expected to show a high retention rate in meat substitutes. Palm oil has relatively lower levels of unsaturation than other oils because it is not subjected to hydrogenation and can, therefore, exist as a solid at room temperature [19]. Palm oil can be effective in improving the retention and succulent properties of meat analogs.

The quality of meat analog deteriorates during freeze–thaw cycles [20]. Moreover, Toth et al. [21] reported that meals prepared with meat analogs had increased food safety risk compared to the meals prepared with natural agents. Nevertheless, only a few studies have been reported on the long-term stability of meat analogs stored under frozen conditions.

This study was conducted to prepare meat analogs supplemented with vegetable oils and evaluate the changes in physicochemical characteristics and quality of the meat analog under different storage conditions of temperature and time.

## 2. Materials and Methods

### 2.1. Materials

Soy protein isolate (SPI, Avention, Incheon, Republic of Korea), textured vegetable protein (TVP, Supromax 5050^®^, and Supromax 5010^®^, DuPont Korea, Seoul, Republic of Korea), and binder (Meatline^®^ 2714, Danisco, Copenhagen, Denmark) were used as a base for the meat analog. SPI contains 90% protein on a dry matter basis. TVP contains 55–60% SPI and 40–45% wheat gluten and wheat starch. Furthermore, the binder is a mixture of egg white powder, glucose, soy protein, locust bean gum, carrageenan, and guar gum (supplied information by DuPont Korea). The meat analog was produced by adding palm oil (Lottefoods, Cheonan, Republic of Korea), orange oil (Sigma-Aldrich Inc., St. Louis, MO, USA), castor oil (Daejung Chemicals, Siheung, Gyeonggi, Republic of Korea), shortening (Hain Celestial Group, Inc., Lake Success, NY, USA), and margarine (Ottogi, Anyang, Republic of Korea) to the base. All the oils were purchased at the local store.

### 2.2. Sample Preparation

Hundred grams of TVP Supromax 5050^®^ and Supromax 5010^®^ were kept immersed in distilled water (10-times their weight) for 2 h and dehydrated in a dehydrator (WS-6600, Hanil Electric, Seoul, Republic of Korea) for 5 min at 1200 rpm. The dehydrated TVP Supromax 5050^®^ and Supromax 5010^®^ in a 1:2 ratio (100 g), SPI (4.5 g), binder (3 g), and five types of vegetable oils (30 g) were mixed in a blender (Multiquick 3 Vario MQ 3145, Braun, Kronberg im Taunus, Germany) for 90 s (Wi et al., 2020). After preheating at 180 °C, the mixed dough (19 g) was placed in a stainless-steel cylindrical mold and cooked in an oven (M4207, Simfer, Istanbul, Turkey) for 14 min. The cooked meat analog was cooled to room temperature for 30 min before being used for further analysis [15].

### 2.3. Storage Conditions

To confirm the storage stability of the meat analog, the molded dough was stored for 6 months, and its physicochemical properties were analyzed. The sample was placed in a container (17 × 10 × 3 cm) and stored in a freezer (A255WD, LG, Seoul, Republic of Korea) at −18 or −60 °C. For microbial analysis, samples were vacuum-packed using a vacuum packaging machine (TYPE574, Solis, Mendrisio, Swiss). The analysis was performed in view of the possibility of exposure to microorganisms during the manufacturing and molding of the dough. The samples were heat-sterilized at 94 °C for 40 min before storage. The stored samples were thawed at a low temperature (4 °C) for 36 h in a refrigerator (A255WD, LG, Seoul, Republic of Korea) and used for analysis at 1-month intervals.

### 2.4. Experimental Methods

#### 2.4.1. Cooking Loss

The cooking loss was calculated by determining the weight of the meat analog before and after heating using the formula given below [22]. The temperature (80 °C) in the middle of the meat analog sample was measured after heating and cooking. The weight of cooked dough was measured after cooling it at room temperature for 30 min, calculated according to Formula (1).

(1) $$\mathrm{Cooking}\;\mathrm{loss}\;\left(\%\right)=\frac{W_{1}-W_{2}}{W_{1}}\;\times \;100$$

W_1_: Weight of sample before heating (g)

W_2_: Weight of sample after heating (g)

#### 2.4.2. Drip Loss

The thawing loss was calculated by measuring the weight of meat analog before and after thawing using Formula (2):

(2) $$\mathrm{Drip}\;\mathrm{loss}\;\left(\%\right)=\frac{W_{1}-W_{2}}{W_{1}}\;\times \;100$$

W_1_: Weight of sample before thawing (g)

W_2_: Weight of sample after thawing (g)

#### 2.4.3. Water Content

The moisture content was measured by heating and drying under atmospheric pressure by taking 1 g each of meat analog dough and cooked meat analog, according to the Association of Agricultural Chemists [23] method (Formula (3)). For cooked meat analog, the outer part was cut, and measurement was performed using the inner part.

(3) $$\mathrm{Water}\;\mathrm{content}\;\left(\%\right)=\frac{W_{1}-W_{2}}{W_{1}}\;\times \;100$$

W_1_: Weight of sample before drying (g)

W_2_: Weight of sample after drying (g)

#### 2.4.4. Liquid-Holding Capacity

The method described by Lorenzo et al. [24] was used with some modification to simultaneously measure the liquid-holding capacity (LHC) of meat analog dough and cooked meat analog with water and oil retention. Each sample was placed in a 15 mL conical tube containing 1 g sterile gauze and stored overnight at 4, 25, or 35 °C. Thereafter, the samples were centrifuged (Labogene 1736R, GYROZEN Co., Ltd., Kimpo, Republic of Korea) at 3000 rpm for 10 min under each temperature condition. The weight of the samples before and after centrifugation was calculated using Formula (4):

(4) $$\mathrm{Liquid}-\mathrm{holding}\;\mathrm{capacity}\;\left(\%\right)=\frac{W_{1}-W_{2}}{W_{1}}\;\times \;100$$

W_1_: Weight of sample before centrifugation (g)

W_2_: Weight of sample weight after centrifugation (g)

#### 2.4.5. Hardness 

Hardness was measured by modifying a method described previously by Lin et al. [25]. For measuring the hardness of meat analog, the sample was cooled and cut into cubes of 2 cm^3^ volume. Hardness was measured with a texture analyzer (CT3-1000, Brookfield Engineering Laboratory, Inc., Middleboro, MA, USA) using a cylindrical probe (TA4/1000). The measurement conditions were as follows: strain rate, 40%; measurement speed, 2.5 mm/s; trigger load, 10 g. The measurements were repeated 10 times for each treatment group.

#### 2.4.6. Chromaticity

The color of cooked meat analog was measured using a colorimeter (CR-400, Konica Minolta, Inc., Tokyo, Japan), following the company’s instructions. The colorimeter was calibrated using a white standard plate (L* = 94.65, a* = −0.46, b* = 2.87). The brightness (L*), redness (a*), and yellowness (b*) in the center of samples were measured 10 times.

#### 2.4.7. DPPH-Radical-Scavenging Activity

The 2,2-diphenyl-1-picrylhydrazyl (DPPH; Sigma-Aldrich, St. Louis, MO, USA) content of cooked meat analog was measured using a modified Jeong method [26] to determine the free-radical-scavenging activity in the cooked meat analog. One gram of freeze-dried meat analog was extracted with 25 mL of 70% ethanol for 3 h in a water tank (BF-30SB; Biofree, Seoul, Republic of Korea) set at 80 °C. The extract was filtered through Whatman No. 2 filter paper (Healthcare Life Science, Buckinghamshire, UK). The filtrate was concentrated using a reduced pressure concentrator (EYELA rotary evaporator N-1000, SUNILEYELA, Seongnam, Republic of Korea). For using the concentrate as a sample, the concentrate was dried to a powder in a freeze dryer (MCFD8512, Ilshinbiobase Co., Dongducheon, Republic of Korea). The powder was dissolved in distilled water at a concentration of 1 mg/mL and used as a sample. The sample (0.1 mL) was allowed to react with 0.1 mL of 0.2 mM DPPH reagent in the dark for 30 min at room temperature, and the absorbance at 517 nm was measured using a spectrophotometer (Multiskan GO, Thermo Scientific, Waltham, MA, USA). In the control, 0.1 mL methanol was added instead of the sample. To correct the absorbance for the intrinsic color of the sample, methanol was added instead of the DPPH reagent to measure the absorbance using the same method. The DPPH-radical-scavenging activity was calculated by substituting the absorbance values in Formula (5):

(5) $$\mathrm{DPPH}\;\mathrm{radical}-\mathrm{scavenging}\;\mathrm{activity}\left(\%\right)=\;(1\;-\frac{\mathrm{Absorbance}\;\mathrm{with}\;\mathrm{sample}\;\mathrm{addition}\;-\;\mathrm{Absorbance}\;\mathrm{due}\;\mathrm{to}\;\mathrm{intrinsic}\;\mathrm{color}\;\mathrm{of}\;\mathrm{sample}}{\mathrm{Absorbance}\;\mathrm{without}\;\mathrm{sample}\;\mathrm{addition}})\;\times \;100$$

#### 2.4.8. Microbial Analysis

Microbial analysis of the meat analog was measured using the method of the Association of Official Analytical Chemists (AOAC) International [23]. The meat analog dough was heat-sterilized at 94 °C for 40 min. Thereafter, the samples stored for different periods according to the storage conditions were diluted with a sterile 0.85% NaCl solution to measure the total number of bacteria and the number of coliforms. The sample (5 g) was mixed with 45 mL NaCl solution and homogenized for 3 min using a homogenizer. One milliliter of the sample solution was mixed with 9 mL NaCl solution and serially diluted. The diluted sample solutions were inoculated on 3 M Petrifilm (Petrifilm^TM^ plate, 3 M Co., St. Paul, MN, USA) and incubated at 37 ± 1 °C for 48 h, followed by counting of the colonies.

### 2.5. Statistical Analysis

The experiments were performed more than three times. The data were analyzed using SPSS statistics (ver. 24.0, SPSS Inc., Chicago, IL, USA). Differences between samples were verified using one-way ANOVA followed by Duncan’s multiple range test (*p* < 0.05). An independent samples *t*-test was used to compare the means of data for the uncooked and cooked samples.

## 3. Results and Discussion

### 3.1. Cooking Loss

Table 1 shows the effects of temperature and storage period on the cooking loss in vegetable meat analogs supplemented with different vegetable oils. The cooking loss increased with an increase in the storage period irrespective of the type of oil added. The main reason for the increase in cooking loss could be the dissolution of oil during the cooking process. Vieira et al. [27] reported that the denaturation of proteins during cooking results in the weakening of the chemical binding force between the protein and oil, leading to the elution of oil. These results are consistent with those of Bentley et al. [28], who showed that the cooking loss in beef patties prepared in the form of ground meat increased with an increase in the storage period. The difference in heat loss between frozen samples with different storage temperatures is generally less at low temperatures [29,30]. 

Under each condition, the cooking loss in the samples supplemented with orange oil, margarine, and shortening was high; the sample supplemented with shortening exhibited the most significant heat loss regardless of the storage temperature and duration (*p* < 0.05). The cooking loss for the sample to which shortening was added was more than 8.08% higher than that for the sample with the lowest loss after heating under each set of conditions. Among the samples stored at −60 °C for 1 month, the cooking loss was 9.68% higher than that for the sample with the lowest cooking loss. The result obtained for the sample containing orange oil could be due to the low water solubility of limonene, the main component of orange oil, as described above [31]. In the case of margarine and shortening, the force binding the oil and the protein being denatured is relatively weaker due to the increase in the content of saturated fatty acids during hydrogenation, because of which a large amount is eluted from the sample when the protein structure changes under heat [27,32].

### 3.2. Drip Loss

Table 2 shows the changes in drip loss in vegetable meat analogs supplemented with different vegetable oils and stored at varying temperatures for different durations. The drip amount during the thawing process varies depending on the type of food and storage conditions. In general, the greater the degree of tissue damage during freezing and storage, the greater the drip [33]. Moreover, the amount of drip generally increases when food is frozen at a high temperature [34]. However, we observed little difference in drip loss between the samples stored at different temperatures and for different periods. In particular, no significant difference was noted for samples supplemented with different types of oils and stored for more than 4 months. The significant difference in the thawing loss in samples stored for 1, 2, and 3 months is considered to be the result of the small deviation. A similar number of ice crystals of similar sizes was generated in the process of freezing of samples at −18 and −60 °C, and the degree of damage to the soybean protein after storage and thawing was reduced. 

Contrary to our results, Hong et al. [35] and Zhu et al. [36] found that the thawing process was slower than the freezing process due to the difference in heat conduction and thermal diffusion between water and ice in the case of frozen food. In other words, our results showed that thawing conditions had a greater effect on the change in the quality of frozen food. Sakata et al. [37] reported that when pork was frozen at −80 and −20 °C, stored at −20 °C for 1 month, and then thawed at 2 °C, the drip loss for both samples was not significantly different, which is similar to our results. In this experiment, each sample frozen at −18 and −60 °C was thawed at 4 °C for 36 h and used for analysis.

### 3.3. Moisture Content

Table 3 shows the effects of temperature and storage period on the moisture contents of meat analog dough supplemented with vegetable oils and the cooked meat analog. The moisture content of meat analog showed a tendency to slightly decrease with the duration of storage. This may be the result of the loss of moisture in the sample through drips during the freezing and thawing process. However, the decrease in moisture content was small because the overall loss due to drip was not large. The tendency of the moisture content to decrease in this experiment was similar to that observed by Zhang et al. [38], who showed that the moisture content of sausages decreased with the duration of storage. There was almost no difference in the moisture content of samples stored for more than 5 months except for those cooked with castor oil at the fifth month of storage. The sample stored at −60 °C showed slightly higher water content than the one stored at −18 °C. 

Under each storage condition, the difference in moisture content between the meat analog samples prepared by adding oils other than orange oil was insignificant. Based on this result, we speculate that the effect of oil on the moisture content during storage is small. The probable reason for the water content of the sample to which orange oil was added being higher than that of other samples is the relatively larger decrease in the weight of the sample due to the loss of components other than water [39]. On the contrary, it was difficult to ascertain a clear trend regarding the effects of temperature and storage period on the moisture contents of cooked and uncooked samples.

### 3.4. Liquid-Holding Capacity

The LHCs of meat analog dough supplemented with vegetable oils and the cooked meat analog stored at varying temperatures for different durations were measured (Table 4, Table 5 and Table 6). The LHC showed a tendency to decrease with the storage period and was slightly higher in the sample stored at −60 °C than in the sample stored at −18 °C. These results are similar to those reported by Heo et al. [40], who showed that there was no difference in the LHC of ducks frozen and stored at −50 and −20 °C. The melting point of the sample prepared by adding palm oil, shortening, and margarine was lower than that of each oil regardless of the temperature and storage period; thus, the sample exhibited high liquid retention in the solid state at 4 °C. At 25 and 35 °C, in which cases the samples became liquid, the LHC was low. This trend became more pronounced with an increase in the storage period. In particular, when a sample stored for 6 months at −60 °C with the addition of shortening was cooked, the difference in liquid retention at 4 and 35 °C was 11.03%, which was the largest decrease. According to Tirado-Kulieva et al. [41], the ice crystals formed upon the freezing of food destroy the proteins, thereby causing increased dripping during the thawing process, which reduces the LHC of food. On the contrary, palm oil, shortening, and margarine have high contents of saturated fat and, thus, have relatively weaker binding to the protein [42]. This could be the reason for the low LHC in the liquid state.

### 3.5. Texture Measurement

**Figure 1:**
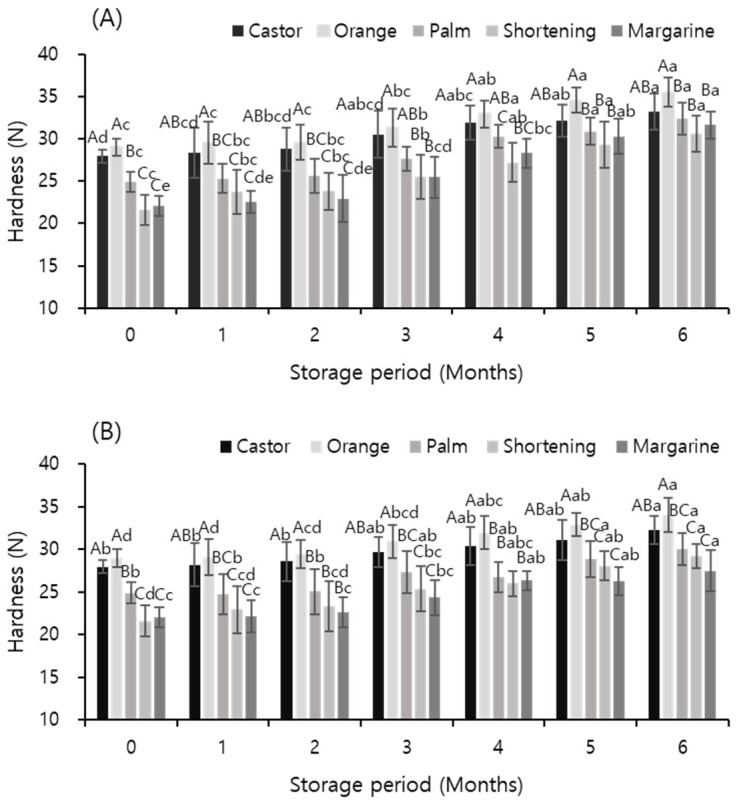
Effects of temperature and storage period on the hardness of meat analogs supplemented with different vegetable oils at −18 °C (**A**) and −60 °C (**B**). ^A–C^ indicates significant differences of means within the oils (*p* < 0.05); ^a–e^ indicates significant differences of means within the storage periods (*p* < 0.05).

Figure 1 shows the effects of temperature and storage period on the hardness of meat analog supplemented with vegetable oils, as determined by measuring the texture. Hardness is an indicator of the age of food [43] and plays an important role in judging the texture of plant meat. The hardness of plant meat increased with the storage period, which is the opposite of the change in hardness of general meat with the storage period. In the case of meat, the ice crystals generated during the freezing process damage the protein tissue, causing the aging of meat; thus, the hardness decreases with the period of storage [44,45]. However, the sample in this study was a plant food prepared with soybean protein as the main material, and unlike meat, it did not have a hard texture and muscle fibers. Therefore, the effect of damage to the protein tissue did not appear as a decrease in hardness. However, the weakening of the binding force between the oil and protein causes the amount of oil eluted during cooking to increase, which ultimately increases the hardness of the sample. In addition, protein damage continues with the increase in storage period, and the amount of oil eluted during cooking further increases.

In general, the quality of preservation increases with a decrease in the storage temperature of frozen food [46]. In this study, the change in the hardness of the sample stored at −60 °C was slightly smaller than that in the hardness of the sample stored at −18 °C. This may be due to the relatively lesser damage to the protein in the sample stored at −60 °C. The results of this experiment were similar to those of Shin et al. [47], who showed that the hardness of garlic stored for 16 months at −18 and −40 °C did not show any significant change during the storage period. In addition, Park et al. [48] reported that the quality of vegetables can be maintained for a longer period than that of meat stored in a frozen state. For different temperatures and periods of storage, the hardness of the sample supplemented with orange oil was the highest under each condition, and the increase in hardness was relatively smaller than that in the samples supplemented with other oils. In particular, when stored at −18 °C for 6 months, the hardness of the sample supplemented with margarine increased by 9.55 N compared to the hardness before storage, whereas the hardness of the sample supplemented with orange oil increased by 6.48 N compared to that before storage. These results indicate that by adding orange oil to meat analog, a chewy product with little change in quality and hardness could be prepared.

### 3.6. Chromaticity

Table 7 and Table 8 show the changes in the colors of vegetable meat analogs supplemented with different vegetable oils and stored at varying temperatures for different durations. The brightness and yellowness of meat analog decreased with the storage period, and the change in the color of the sample stored at −60 °C occurred slower than that in the color of the sample stored at −18 °C. The brightness hardly changed until 3 months of storage, regardless of the storage temperature and type of oil, and started to gradually decrease from 4 months onward. Yellowness also showed a tendency similar to that of brightness. The yellowness did not change until 2 months of storage and gradually decreased from 3 months of storage. The results of this experiment were similar to those of Kim et al. [39], who showed that the brightness and yellowness of leeks stored frozen tended to decrease with the storage period. Among the samples to which different types of oil were added, the brightness and yellowness of the sample to which orange oil was added were significantly high (*p* < 0.05) irrespective of the temperature and storage period. This may be due to the intrinsic color of the orange oil. In a study by Park et al. [49], the color of the oil itself was darker than that of the product, because of which it showed a similar tendency to the decrease in the brightness of the product. On the contrary, no change in redness was observed with different types of oil, storage temperatures, and storage periods.

The color of samples is considered to be different when the value of color difference (ΔE) is 3.0 or more [50]. As shown in Table 8, except for the samples to which margarine was added, the color difference showed a tendency to increase with an increase in the storage period. Moreover, according to the standards of KFII, no color difference was evident between samples assessed immediately after preparation and those assessed before the fourth month of storage. The color difference was noticeable only in some of the samples during the fifth month of storage. The results of this experiment are consistent with those of Lee et al. [51], who reported that the color difference of tofu increased with an increase in the storage period, irrespective of the storage temperature. On the contrary, when margarine was added, the color difference of the sample was comparatively higher than that of the samples to which other oils were added, except for the samples stored for 5 or 6 months. There was a difference in the color of the samples immediately after preparation and after storage for 1 month; the color difference decreased from the second month onward. These results show that the color change appears relatively quickly within 2 months when plant meat is supplemented with margarine.

### 3.7. DPPH-Radical-Scavenging Activity

**Figure 2:**
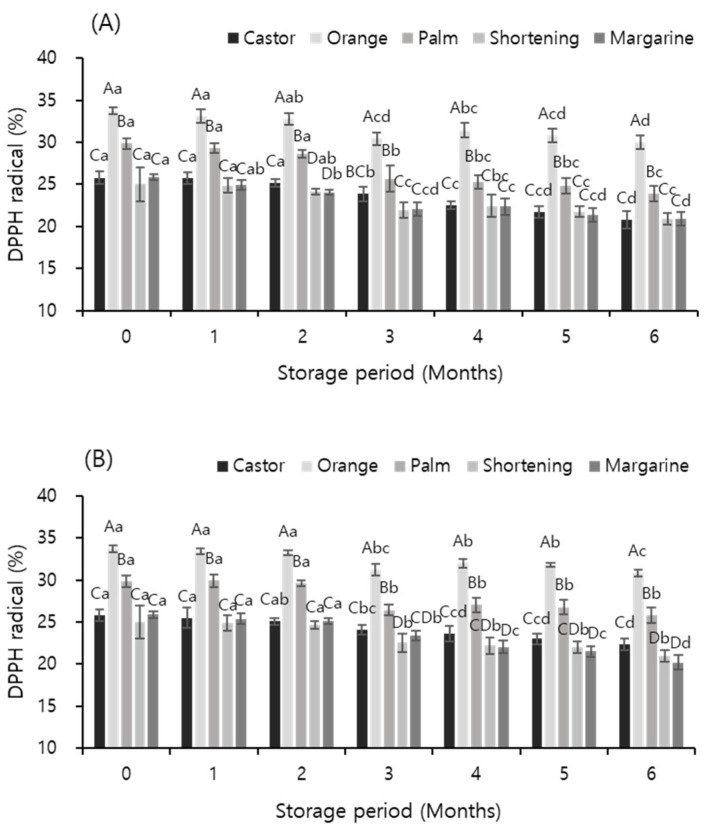
Effects of temperature and storage period on the DPPH-radical-scavenging activity (%) of meat analogs supplemented with different vegetable oils at −18 °C (**A**) and −60 °C (**B**). ^A–D^ indicates significant differences of means within the oils (*p* < 0.05); ^a–d^ indicates significant differences of means within the storage period (*p* < 0.05).

Figure 2 shows the effects of temperature and storage period on the DPPH radical-scavenging activity of meat analog supplemented with vegetable oils. The scavenging activity decreased with an increase in the storage period; the scavenging activity of the sample supplemented with orange oil was the highest irrespective of the storage temperature and period (*p* < 0.05). The scavenging activity of the samples stored at −60 °C was slightly higher than that of the samples stored at −18 °C. The results of this experiment were consistent with those of Jin et al. [52], who showed that the DPPH-radical-scavenging activity of apples decreased with an increase in the storage period. Additionally, the sample to which orange oil was added showed 33.74% scavenging activity immediately after preparation; after storage at −18 and −60 °C for 6 months, its scavenging activity was decreased by 3.73% and 2.87% to 30.01% and 30.87%, respectively. The scavenging activity of the samples to which orange oil was added was 30% or more until the sixth month of storage, but all the samples supplemented with other oils showed scavenging activities of 30% or less. These results indicate the excellent antioxidant activity of orange oil, which has been known for a long time. Vanamala et al. [53] and Yang et al. [54] reported that the peel of citrus fruits contains high amounts of flavonoids, terpenes, vitamins, carotenoids, organic acids, and pectin. In addition, these ingredients exhibit antioxidant, antibacterial, anti-inflammatory, antimutation, antiviral, and capillary-strengthening effects and prevent diseases of the circulatory system [55,56,57].

### 3.8. Microbial Analysis

The changes in the microbial composition of meat analog dough supplemented with vegetable oils and stored at varying temperatures for different durations were assessed as total cell count and the count of coliform group bacteria (Table 9). These bacterial counts were determined by applying the frozen food manufacturing and processing standards announced in the Food Code. The total cell count and the count of coliform group bacteria in frozen foods are described in the Food Code as 5.00 log CFU/g and 1.00 log CFU/g or less, respectively [58]. In this study, the total cell count showed a tendency to increase with an increase in the storage period; the sample stored at −18 °C showed a slightly higher total cell count than that stored at −60 °C. This trend is the same as reported in many previous studies—the total cell count increases with an increase in the storage period, and the growth of microorganisms is inhibited with a decrease in the storage temperature [59,60,61]. The total cell count was not detected immediately after the sample was prepared, vacuum-packed, and heat sterilized at 94 °C for 40 min. The total cell count in the sample after 4 months of storage was 3.18–3.84 log CFU/g, which is within the acceptable range. The coliform group bacteria were not detected until the fourth month of storage. These results indicate that the stability of microorganisms can be secured for up to 4 months if the sample is vacuum-packed and subjected to heat sterilization. These results are similar to those of Roh [62], who reported that no coliform group bacterium was detected in liquid-heated tofu after the heating process. In addition, the total cell count in the sample to which orange oil was added was relatively lower, which is believed to reflect the antibacterial property of orange oil [63].

## 4. Conclusions

In the present study, we evaluated the physicochemical and storage stability properties of meat analogs supplemented with different vegetable oils and stored under cold conditions. Overall, storage temperature including −18 or −60 °C only had a small impact on quality of the meat analogs added with all the oils. Although the quality decreased with an increase in the storage period with regard to the liquid-holding capacity and hardness, the overall change in quality was insignificant. The quality of plant meat stored in a frozen state did not significantly deteriorate until 6 months, as evidenced by the analyses of fat rancidity and microorganisms, which are directly related to stability. Since vegetable oils were added to improve the juiciness and tenderness of meat analogs, the rancidity of meat analogs was also important. For 6 months, only 2.87 to 5.94% of their DPPH radical-scavenging activities were decreased in all the samples. Especially, the meat analog supplemented with orange oil highly maintained its liquid-holding capacity, water content, hardness, and DPPH radical-scavenging activity for at least 6 months, which means that the succulence and texture of meat analog were maintained through frozen storage. 

The results of our study have thus effectively analyzed and presented the physicochemical analyses and storage stability of meat analog added to different oils. The meat analog is still limited to vegetarian consumption; however, these continued studies will exceed the limit in the near future. 

## Figures and Tables

**Table 1:** Effects of temperature and storage period on cooking loss (%) of meat analogs supplemented with different vegetable oils.

ST ^(1)^	Oil	Storage Period (Months)						
		0	1	2	3	4	5	6
−18	Castor oil	8.54 ± 0.65 ^Dcd^	8.17 ± 0.92 ^Ed^	8.46 ± 1.19 ^Dcd^	9.10 ± 0.86 ^Dbc^	9.44 ± 0.98 ^Dab^	9.64 ± 1.15 ^Dab^	10.24 ± 0.90 ^Da^
	Orange oil	13.15 ± 0.74 ^Cc^	13.04 ± 1.12 ^Cc^	13.67 ± 0.86 ^Cbc^	14.45 ± 0.81 ^Cab^	14.55 ± 1.01 ^Ca^	14.83 ± 1.20 ^Ca^	14.96 ± 0.80 ^Ca^
	Palm oil	8.03 ± 0.68 ^Db^	9.18 ± 1.40 ^Da^	9.17 ± 1.27 ^Da^	9.74 ± 1.23 ^Da^	9.67 ± 0.90 ^Da^	9.92 ± 0.97 ^Da^	10.16 ± 1.11 ^Da^
	Shortening	16.71 ± 0.66 ^Ad^	16.91 ± 1.47 ^Acd^	17.05 ± 0.99 ^Abcd^	17.91 ± 1.17 ^Aab^	17.84 ± 0.83 ^Aabc^	17.96 ± 1.14 ^Aab^	18.24 ± 0.88 ^Aa^
	Margarine	14.51 ± 0.85 ^Bc^	15.66 ± 0.87 ^Bb^	15.59 ± 0.86 ^Bb^	16.12 ± 1.44 ^Bab^	16.50 ± 1.16 ^Bab^	16.82 ± 1.31 ^Ba^	17.11 ± 0.98 ^Ba^
−60	Castor oil	8.54 ± 0.65 ^Da^	7.50 ± 0.80 ^Eb^	7.70 ± 1.35 ^Eb^	8.61 ± 0.95 ^Da^	8.97 ± 0.89 ^Da^	8.83 ± 1.16 ^Da^	9.11 ± 0.64 ^Da^
	Orange oil	13.15 ± 0.74 ^Cd^	13.28 ± 1.22 ^Ccd^	13.61 ± 0.72 ^Cbcd^	14.13 ± 1.11 ^Cabc^	13.88 ± 1.16 ^Cabcd^	14.31 ± 0.81 ^Cab^	14.72 ± 1.17 ^Ca^
	Palm oil	8.03 ± 0.68 ^Dc^	8.36 ± 0.72 ^Dc^	8.72 ± 1.07 ^Dbc^	9.29 ± 1.02 ^Dab^	9.51 ± 1.07 ^Dab^	9.35 ± 1.19 ^Dab^	9.69 ± 0.84 ^Da^
	Shortening	16.71 ± 0.66 ^Ab^	17.17 ± 0.94 ^Aab^	16.70 ± 0.94 ^Ab^	17.49 ± 0.86 ^Aab^	17.63 ± 0.85 ^Aa^	17.55 ± 1.16 ^Aab^	17.89 ± 0.96 ^Aa^
	Margarine	14.51 ± 0.85 ^Bc^	14.67 ± 0.93 ^Bc^	14.86 ± 0.85 ^Bbc^	15.42 ± 0.90 ^Bab^	15.58 ± 0.70 ^Bab^	15.87 ± 0.96 ^Ba^	16.05 ± 0.57 ^Ba^

^(1)^ ST, storage temperature (°C). ^A–E^ Means within a column with different letters are significantly different (*p* < 0.05). ^a–d^ Means within a row with different letters are significantly different (*p* < 0.05).

**Table 2:** Effects of temperature and storage period on drip loss (%) in meat analogs supplemented with different vegetable oils.

ST ^(1)^	Oil	Storage Period (Months)					
		1	2	3	4	5	6
−18	Castor oil	0.66 ± 0.15 ^Aa^	0.69 ± 0.20 ^Aa^	0.62 ± 0.19 ^Aa^	0.65 ± 0.34 ^Aa^	0.68 ± 0.32 ^Aa^	0.71 ± 0.26 ^Aa^
	Orange oil	0.57 ± 0.27 ^ABa^	0.61 ± 0.36 ^ABa^	0.58 ± 0.24 ^Aa^	0.64 ± 0.36 ^Aa^	0.67 ± 0.38 ^Aa^	0.64 ± 0.34 ^Aa^
	Palm oil	0.45 ± 0.19 ^BCa^	0.40 ± 0.17 ^Ba^	0.47 ± 0.17 ^Aa^	0.52 ± 0.18 ^Aa^	0.51 ± 0.21 ^Aa^	0.54 ± 0.26 ^Aa^
	Shortening	0.42 ± 0.12 ^Ca^	0.49 ± 0.23 ^ABa^	0.48 ± 0.22 ^Aa^	0.53 ± 0.22 ^Aa^	0.50 ± 0.36 ^Aa^	0.58 ± 0.39 ^Aa^
	Margarine	0.44 ± 0.08 ^BCa^	0.42 ± 0.27 ^Ba^	0.51 ± 0.24 ^Aa^	0.56 ± 0.27 ^Aa^	0.53 ± 0.26 ^Aa^	0.51 ± 0.20 ^Aa^
−60	Castor oil	0.56 ± 0.19 ^Aa^	0.61 ± 0.25 ^Aa^	0.62 ± 0.15 ^Aa^	0.66 ± 0.41 ^Aa^	0.62 ± 0.32 ^Aa^	0.65 ± 0.24 ^Aa^
	Orange oil	0.53 ± 0.20 ^ABa^	0.59 ± 0.14 ^Aa^	0.60 ± 0.26 ^ABa^	0.64 ± 0.35 ^Aa^	0.63 ± 0.38 ^Aa^	0.62 ± 0.29 ^Aa^
	Palm oil	0.41 ± 0.16 ^Ba^	0.49 ± 0.20 ^ABa^	0.44 ± 0.13 ^Ba^	0.53 ± 0.40 ^Aa^	0.49 ± 0.18 ^Aa^	0.54 ± 0.14 ^Aa^
	Shortening	0.43 ± 0.12 ^ABa^	0.45 ± 0.15 ^ABa^	0.49 ± 0.21 ^ABa^	0.54 ± 0.25 ^Aa^	0.51 ± 0.38 ^Aa^	0.50 ± 0.31 ^Aa^
	Margarine	0.39 ± 0.18 ^Ba^	0.37 ± 0.12 ^Ba^	0.48 ± 0.18 ^ABa^	0.50 ± 0.20 ^Aa^	0.47 ± 0.21 ^Aa^	0.51 ± 0.29 ^Aa^

^(1)^ ST, storage temperature (°C). ^A–C^ Means within a column with different letters are significantly different (*p* < 0.05). ^a^ Means within a row with different letters are significantly different (*p* < 0.05).

**Table 3:** Effects of temperature and storage period on the water content (%) of meat analogs supplemented with different vegetable oils.

Cooking Status	ST ^(1)^	Oil	Storage Period (Months)						
			0	1	2	3	4	5	6
Cooking	−18	Castor oil	49.81 ± 0.31 ^Ca^	51.14 ± 0.97 ^BCa^	50.55 ± 0.29 ^Ba^	48.85 ± 0.40 ^Bb^	48.67 ± 0.40 ^Bb^	49.91 ± 0.45 ^Bb^	47.11 ± 0.62 ^Bb^
		Orange oil	67.70 ± 0.30 ^Abc^	69.10 ± 0.39 ^Aa^	68.54 ± 0.58 ^Aab^	66.71 ± 1.02 ^Acd^	66.81 ± 0.45 ^Acd^	65.52 ± 0.33 ^Acd^	64.85 ± 0.91 ^Ad^
		Palm oil	50.07 ± 0.54 ^Ca^	52.91 ± 0.92 ^Ba^	50.82 ± 0.62 ^Bab^	49.25 ± 0.94 ^Bab^	49.42 ± 0.55 ^Bab^	48.34 ± 0.78 ^Cab^	47.81 ± 0.85 ^Bb^
		Shortening	51.26 ± 0.80 ^Ba^	50.04 ± 1.59 ^Cb^	49.10 ± 0.65 ^Cb^	50.42 ± 0.97 ^Bb^	49.33 ± 0.35 ^Bb^	48.84 ± 0.54 ^BCb^	48.26 ± 0.72 ^Bb^
		Margarine	51.12 ± 0.46 ^Ba^	51.26 ± 1.15 ^BCb^	49.51 ± 0.39 ^Cbc^	49.04 ± 0.50 ^Bbc^	48.76 ± 0.93 ^Bbc^	48.37 ± 0.76 ^Cbc^	47.85 ± 0.86 ^Bc^
	−60	Castor oil	49.81 ± 0.31 ^Cab^	51.90 ± 1.62 ^Ba^	50.77 ± 0.98 ^Bab^	49.47 ± 0.68 ^Babc^	49.53 ± 0.49 ^Babc^	49.32 ± 0.80 ^Bbc^	48.92 ± 0.90 ^Bc^
		Orange oil	67.70 ± 0.30 ^Aabc^	68.42 ± 1.02 ^Aa^	68.72 ± 0.52 ^Aab^	67.20 ± 0.61 ^Abc^	66.70 ± 0.44 ^Abc^	66.58 ± 0.55 ^Ac^	66.07 ± 0.74 ^Ac^
		Palm oil	50.07 ± 0.54 ^Ca^	50.23 ± 1.05 ^Bab^	50.51 ± 0.78 ^Babc^	49.64 ± 0.41 ^Babc^	49.26 ± 0.80 ^Babc^	48.83 ± 0.27 ^Bbc^	48.55 ± 0.53 ^Bc^
		Shortening	51.26 ± 0.80 ^Ba^	50.79 ± 1.33 ^Ba^	49.73 ± 0.96 ^Bb^	49.88 ± 0.90 ^Bb^	49.60 ± 0.59 ^Bb^	49.24 ± 0.54 ^Bb^	48.94 ± 0.78 ^Bb^
		Margarine	51.12 ± 0.46 ^Ba^	51.37 ± 0.98 ^Bab^	50.64 ± 0.43 ^Bbc^	49.25 ± 0.58 ^Bbc^	49.50 ± 0.50 ^Bbc^	49.09 ± 0.24 ^Bbc^	48.74 ± 0.41 ^Bc^
Non-cooking	−18	Castor oil	50.17 ± 0.34 ^Bb^	51.18 ± 1.13 ^Ba^	50.18 ± 0.60 ^Bab^	48.21 ± 0.67 ^Cc^	48.30 ± 0.67 ^Bc^	48.16 ± 0.86 ^Bb^	47.61 ± 1.01 ^Bd^
		Orange oil	67.66 ± 1.43 ^Abc^	69.24 ± 0.88 ^Aa^	68.63 ± 0.32 ^Aab^	66.47 ± 0.57 ^Ac^	66.95 ± 0.32 ^Ac^	66.26 ± 0.63 ^Ad^	65.76 ± 0.74 ^Ad^
		Palm oil	50.49 ± 0.80 ^Bbc^	50.43 ± 1.99 ^Ba^	49.53 ± 0.49 ^Bb^	49.86 ± 0.54 ^Bcd^	49.32 ± 0.85 ^Bcd^	49.06 ± 0.45 ^Bde^	48.45 ± 0.31 ^Be^
		Shortening	51.45 ± 1.17 ^Ba^	49.18 ± 1.07 ^Bab^	49.36 ± 0.80 ^Bbc^	49.75 ± 0.72 ^Bab^	49.24 ± 0.98 ^Bbc^	48.91 ± 0.74 ^Bbc^	48.44 ± 0.85 ^Bc^
		Margarine	52.35 ± 1.52 ^Ba^	50.10 ± 0.92 ^Ba^	49.75 ± 0.39 ^Bb^	49.47 ± 0.51 ^Bbc^	49.16 ± 0.28 ^Bbc^	48.59 ± 0.98 ^Bbc^	48.14 ± 1.08 ^Bc^
	−60	Castor oil	50.17 ± 0.34 ^Bbc^	50.60 ± 1.23 ^Ba^	50.23 ± 0.85 ^Bab^	49.19 ± 0.65 ^Bbc^	49.58 ± 0.46 ^Bbc^	48.86 ± 0.65 ^Bbc^	48.49 ± 0.75 ^Bc^
		Orange oil	67.66 ± 1.43 ^Aab^	69.38 ± 1.44 ^Aa^	68.81 ± 0.68 ^Aa^	67.51 ± 0.80 ^Abc^	67.10 ± 0.33 ^Abc^	66.91 ± 0.71 ^Abc^	66.58 ± 0.95 ^Ac^
		Palm oil	50.49 ± 0.80 ^Bab^	50.35 ± 1.12 ^Ba^	49.72 ± 0.56 ^Ba^	49.92 ± 0.21 ^Babc^	49.84 ± 0.67 ^Babc^	49.15 ± 0.42 ^Bbc^	48.85 ± 0.67 ^Bc^
		Shortening	51.45 ± 1.17 ^Ba^	51.22 ± 0.88 ^Bab^	49.28 ± 0.84 ^Babc^	49.41 ± 0.67 ^Babc^	49.28 ± 0.63 ^Babc^	49.10 ± 0.55 ^Bbc^	48.69 ± 0.64 ^Bc^
		Margarine	52.35 ± 1.52 ^Ba^	51.16 ± 1.23 ^Ba^	50.15 ± 0.41 ^Ba^	49.59 ± 0.91 ^Bb^	49.63 ± 0.95 ^Bb^	49.40 ± 0.73 ^Bb^	48.98 ± 0.78 ^Bb^

^(1)^ ST, storage temperature (°C). ^A–C^ Means within a column with different letters are significantly different (*p* < 0.05). ^a–e^ Means within a row with different letters are significantly different (*p* < 0.05).

**Table 4:** Effects of temperature and storage period on the liquid-holding capacity (%) of meat analogs supplemented with different vegetable oils at 4 °C.

Cooking Status	ST ^(1)^	Oil	Storage Period (Months)						
			0	1	2	3	4	5	6
Cooking	−18	Castor oil	92.99 ± 0.57 ^Ca^	91.11 ± 0.83 ^Db^	90.97 ± 0.62 ^Cbc^	90.27 ± 0.73 ^Cbcd^	89.74 ± 0.82 ^Bcd^	89.05 ± 0.55 ^Bde^	87.98 ± 0.84 ^Be^
		Orange oil	96.79 ± 0.61 ^Aa^	96.01 ± 0.29 ^Aab^	95.66 ± 0.89 ^Abc^	94.89 ± 0.75 ^Ac^	93.46 ± 0.44 ^Ad^	92.91 ± 0.46 ^Ade^	92.09 ± 0.27 ^Ae^
		Palm oil	95.33 ± 0.47 ^Ba^	94.12 ± 0.57 ^Cab^	93.81 ± 0.97 ^Bbc^	92.82 ± 0.65 ^Bcd^	92.52 ± 0.91 ^Ad^	91.87 ± 0.28 ^Ade^	91.08 ± 0.75 ^Ae^
		Shortening	95.62 ± 0.41 ^Ba^	94.46 ± 0.48 ^BCb^	94.10 ± 0.89 ^ABbc^	93.27 ± 0.72 ^Bcd^	93.17 ± 0.73 ^Acde^	92.82 ± 0.56 ^Ade^	92.03 ± 0.51 ^Ae^
		Margarine	96.11 ± 0.82 ^ABa^	95.19 ± 0.32 ^ABab^	94.52 ± 0.98 ^ABbc^	93.70 ± 0.58 ^ABcd^	93.39 ± 0.49 ^Acd^	92.55 ± 0.90 ^Ad^	91.01 ± 0.43 ^Ae^
	−60	Castor oil	92.99 ± 0.57 ^Ca^	91.61 ± 0.68 ^Cb^	91.28 ± 0.96 ^Bb^	90.57 ± 0.85 ^Bbc^	89.67 ± 1.06 ^Bcd^	88.84 ± 0.71 ^Bde^	87.95 ± 0.49 ^Ce^
		Orange oil	96.79 ± 0.61 ^Aa^	96.56 ± 0.42 ^Aa^	96.24 ± 0.94 ^Aab^	95.58 ± 0.86 ^Aab^	94.83 ± 0.95 ^Abc^	94.14 ± 0.93 ^Acd^	93.03 ± 0.44 ^Ad^
		Palm oil	95.33 ± 0.47 ^Ba^	95.18 ± 0.42 ^Ba^	94.92 ± 0.83 ^Aa^	94.21 ± 0.80 ^Aab^	93.49 ± 0.67 ^Abc^	92.93 ± 0.68 ^Acd^	91.98 ± 0.34 ^Bd^
		Shortening	95.62 ± 0.41 ^Ba^	95.54 ± 0.48 ^Ba^	95.12 ± 0.82 ^Aa^	94.42 ± 0.50 ^Aab^	93.83 ± 0.93 ^Ab^	93.23 ± 0.85 ^Ab^	92.03 ± 0.59 ^Bc^
		Margarine	96.11 ± 0.82 ^ABa^	96.03 ± 0.36 ^ABa^	95.57 ± 0.75 ^Aab^	94.86 ± 0.52 ^Aabc^	94.35 ± 0.81 ^Abc^	93.98 ± 0.79 ^Acd^	92.99 ± 0.47 ^Ad^
Non-cooking	−18	Castor oil	91.39 ± 0.86 ^Ca^	91.82 ± 1.18 ^Ca^	91.31 ± 0.69 ^Ba^	90.49 ± 0.57 ^Cab^	89.40 ± 0.90 ^Cbc^	88.78 ± 0.69 ^Ccd^	87.90 ± 0.53 ^Cd^
		Orange oil	97.21 ± 0.13 ^Aa^	96.85 ± 0.60 ^Aab^	96.63 ± 0.97 ^Aab^	95.86 ± 0.66 ^Abc^	95.30 ± 0.82 ^Ac^	94.87 ± 0.35 ^Acd^	94.02 ± 0.60 ^Ad^
		Palm oil	95.94 ± 0.31 ^Ba^	95.04 ± 0.58 ^Bab^	94.81 ± 0.93 ^Aabc^	94.39 ± 0.70 ^Bbcd^	93.67 ± 0.68 ^Bcd^	93.27 ± 0.68 ^Bd^	91.97 ± 0.85 ^Be^
		Shortening	96.58 ± 0.24 ^ABa^	96.26 ± 0.97 ^ABab^	95.89 ± 0.88 ^Aab^	95.08 ± 0.95 ^ABbc^	94.47 ± 0.59 ^ABc^	93.95 ± 0.44 ^ABcd^	92.92 ± 0.61 ^ABd^
		Margarine	95.70 ± 0.97 ^Bab^	95.94 ± 0.78 ^ABa^	95.66 ± 1.28 ^Aab^	94.92 ± 0.51 ^ABabc^	94.14 ± 1.02 ^ABbc^	93.61 ± 0.79 ^Bc^	92.05 ± 0.53 ^Bd^
	−60	Castor oil	91.39 ± 0.86 ^Ca^	91.61 ± 0.91 ^Ca^	91.31 ± 0.94 ^Ca^	90.74 ± 0.48 ^Cab^	90.38 ± 0.58 ^Cabc^	89.76 ± 0.84 ^Cbc^	89.03 ± 0.34 ^Cc^
		Orange oil	97.21 ± 0.13 ^Aa^	97.17 ± 0.20 ^Aa^	96.96 ± 0.51 ^Aab^	96.35 ± 0.56 ^Aabc^	95.88 ± 0.99 ^Abc^	95.32 ± 0.52 ^Ac^	94.05 ± 0.93 ^Ad^
		Palm oil	95.94 ± 0.31 ^Ba^	95.55 ± 0.38 ^Bab^	95.22 ± 0.96 ^Bab^	94.53 ± 0.80 ^Bbc^	93.85 ± 0.66 ^Bc^	93.53 ± 0.38 ^Bc^	91.95 ± 0.53 ^Bd^
		Shortening	96.58 ± 0.24 ^ABa^	96.06 ± 0.71 ^ABab^	95.68 ± 0.96 ^ABabc^	95.23 ± 0.42 ^Bbcd^	94.58 ± 0.54 ^ABcd^	94.12 ± 0.72 ^ABd^	92.99 ± 0.47 ^ABe^
		Margarine	95.70 ± 0.97 ^Bab^	96.20 ± 0.62 ^ABa^	95.91 ± 0.92 ^ABab^	95.01 ± 0.59 ^Babc^	94.64 ± 0.64 ^ABbc^	93.86 ± 0.96 ^Bc^	92.02 ± 0.61 ^Bd^

^(1)^ ST, storage temperature (°C). ^A–D^ Means within a column with different letters are significantly different (*p* < 0.05). ^a–e^ Means within a row with different letters are significantly different (*p* < 0.05).

**Table 5:** Effects of temperature and storage period on the liquid-holding capacity (%) of meat analogs supplemented with different vegetable oils at 25 °C.

Cooking Status	ST ^(1)^	Oil	Storage Period (Months)						
			0	1	2	3	4	5	6
Cooking	−18	Castor oil	91.56 ± 0.86 ^Ba^	89.84 ± 0.98 ^Bb^	89.34 ± 1.11 ^Bb^	87.83 ± 0.84 ^Bc^	87.34 ± 0.64 ^Bcd^	86.90 ± 0.59 ^Bcd^	86.12 ± 0.53 ^Bd^
		Orange oil	96.32 ± 1.01 ^Aa^	95.74 ± 0.94 ^Aa^	95.37 ± 0.76 ^Aa^	93.89 ± 0.63 ^Ab^	93.67 ± 0.84 ^Ab^	93.19 ± 0.62 ^Abc^	92.06 ± 0.56 ^Ac^
		Palm oil	87.18 ± 0.88 ^Da^	86.93 ± 0.71 ^Ca^	86.52 ± 1.05 ^Ca^	84.34 ± 0.71 ^Cb^	83.87 ± 0.76 ^Db^	83.23 ± 0.34 ^Dbc^	82.05 ± 0.83 ^Ec^
		Shortening	90.09 ± 0.68 ^BCa^	89.86 ± 0.77 ^Ba^	88.62 ± 0.62 ^Bb^	86.78 ± 0.80 ^Bc^	86.42 ± 0.70 ^BCc^	85.91 ± 0.52 ^BCcd^	85.07 ± 0.48 ^Cd^
		Margarine	89.30 ± 0.92 ^Ca^	88.67 ± 1.09 ^Ba^	88.34 ± 0.49 ^Ba^	86.48 ± 0.67 ^Bb^	85.82 ± 0.56 ^Cb^	85.22 ± 0.65 ^Cbc^	84.02 ± 0.23 ^Dc^
	−60	Castor oil	91.56 ± 0.86 ^Ba^	90.08 ± 0.59 ^Bb^	89.71 ± 0.81 ^Bb^	88.19 ± 0.46 ^Bc^	87.70 ± 0.89 ^Bc^	86.92 ± 0.89 ^Bcd^	86.05 ± 0.47 ^Bd^
		Orange oil	96.32 ± 1.01 ^Aa^	96.08 ± 0.20 ^Aa^	95.79 ± 0.66 ^Aa^	94.11 ± 0.76 ^Ab^	94.19 ± 0.94 ^Ab^	93.84 ± 0.95 ^Ab^	93.06 ± 0.33 ^Ab^
		Palm oil	87.18 ± 0.88 ^Da^	87.51 ± 0.95 ^Ca^	87.18 ± 0.79 ^Ca^	85.42 ± 0.80 ^Cb^	84.71 ± 0.80 ^Cb^	84.22 ± 0.55 ^Cbc^	83.15 ± 0.31 ^Dc^
		Shortening	90.09 ± 0.68 ^BCa^	90.04 ± 0.40 ^Ba^	89.29 ± 0.77 ^Ba^	86.98 ± 0.77 ^Bb^	86.35 ± 0.52 ^Bb^	85.93 ± 0.72 ^Bbc^	84.98 ± 0.47 ^Cc^
		Margarine	89.30 ± 0.92 ^Ca^	89.74 ± 0.85 ^Ba^	89.22 ± 1.16 ^Ba^	87.19 ± 0.94 ^Bb^	86.55 ± 0.66 ^Bbc^	85.59 ± 0.67 ^BCcd^	84.11 ± 0.77 ^Cd^
Non-cooking	−18	Castor oil	91.33 ± 0.31 ^Ba^	90.32 ± 0.97 ^Bab^	90.03 ± 0.89 ^Bab^	88.95 ± 0.75 ^Bbc^	88.27 ± 0.77 ^Bcd^	87.88 ± 0.63 ^Bcd^	87.02 ± 0.78 ^Be^
		Orange oil	96.71 ± 0.41 ^Aa^	97.02 ± 0.55 ^Aa^	96.39 ± 1.09 ^Aab^	95.35 ± 0.47 ^Abc^	94.94 ± 0.67 ^Acd^	94.19 ± 0.45 ^Ad^	93.05 ± 0.40 ^Ae^
		Palm oil	90.21 ± 0.94 ^Ba^	90.15 ± 0.75 ^Ba^	90.01 ± 0.94 ^Bab^	88.72 ± 0.44 ^Bbc^	88.35 ± 0.81 ^Bc^	87.54 ± 0.73 ^BCc^	86.07 ± 0.45 ^BCd^
		Shortening	90.22 ± 0.78 ^Ba^	90.05 ± 0.80 ^Ba^	89.36 ± 0.87 ^Bab^	87.95 ± 0.82 ^Bbc^	87.22 ± 0.79 ^Bc^	86.58 ± 0.79 ^CDc^	85.03 ± 0.97 ^Cd^
		Margarine	90.69 ± 0.37 ^Ba^	89.97 ± 0.30 ^Ba^	89.68 ± 1.29 ^Ba^	88.38 ± 0.88 ^Bb^	87.61 ± 0.57 ^Bb^	85.55 ± 0.56 ^Dc^	84.97 ± 0.71 ^Cc^
	−60	Castor oil	91.33 ± 0.31 ^Ba^	91.47 ± 0.43 ^Ba^	91.10 ± 0.73 ^Ba^	90.03 ± 0.62 ^Bb^	89.70 ± 0.47 ^Bbc^	88.80 ± 0.46 ^Bcd^	87.95 ± 0.76 ^Bd^
		Orange oil	96.71 ± 0.41 ^Aab^	97.18 ± 0.49 ^Aa^	96.97 ± 0.78 ^Aa^	95.70 ± 0.71 ^Abc^	95.46 ± 0.77 ^Ac^	94.80 ± 0.82 ^Acd^	93.98 ± 0.34 ^Ad^
		Palm oil	90.21 ± 0.94 ^Bab^	90.70 ± 0.51 ^Ba^	90.19 ± 0.96 ^BCab^	88.93 ± 0.75 ^BCbc^	88.25 ± 0.90 ^BCc^	87.58 ± 0.62 ^BCc^	86.03 ± 0.75 ^Cd^
		Shortening	90.22 ± 0.78 ^Ba^	89.56 ± 0.77 ^Ca^	89.17 ± 0.93 ^Cab^	88.03 ± 0.66 ^Cbc^	87.42 ± 1.02 ^Ccd^	86.96 ± 0.73 ^Ccd^	85.97 ± 0.81 ^Cd^
		Margarine	90.69 ± 0.37 ^Bab^	91.26 ± 0.79 ^Ba^	90.67 ± 1.02 ^BCab^	89.32 ± 0.78 ^BCbc^	88.90 ± 0.75 ^BCc^	88.23 ± 0.94 ^BCcd^	86.96 ± 0.46 ^BCd^

^(1)^ ST, storage temperature (°C). ^A–E^ Means within a column with different letters are significantly different (*p* < 0.05). ^a–e^ Means within a row with different letters are significantly different (*p* < 0.05).

**Table 6:** Effects of temperature and storage period on the liquid-holding capacity (%) of meat analogs supplemented with different vegetable oils at 35 °C.

Cooking Status	ST ^(1)^	Oil	Storage Period (Months)						
			0	1	2	3	4	5	6
Cooking	−18	Castor oil	90.99 ± 0.54 ^Ba^	90.65 ± 0.60 ^Ba^	90.27 ± 0.91 ^Ba^	88.29 ± 0.91 ^Bb^	87.52 ± 0.62 ^Bbc^	86.87 ± 0.91 ^Bcd^	85.96 ± 0.31 ^Bd^
		Orange oil	95.26 ± 0.76 ^Aa^	94.74 ± 0.86 ^Aa^	94.48 ± 1.04 ^Aa^	92.66 ± 0.54 ^Ab^	91.94 ± 0.65 ^Ab^	91.29 ± 0.76 ^Abc^	89.97 ± 0.92 ^Ac^
		Palm oil	88.39 ± 0.44 ^Ca^	87.82 ± 0.61 ^Ca^	87.41 ± 0.92 ^Ca^	85.61 ± 0.84 ^Cb^	84.92 ± 0.36 ^Cbc^	84.12 ± 0.98 ^Ccd^	82.99 ± 0.27 ^Cd^
		Shortening	87.24 ± 0.49 ^Ca^	86.28 ± 0.53 ^Da^	86.04 ± 1.07 ^Ca^	84.21 ± 0.78 ^Db^	83.77 ± 0.53 ^Dbc^	82.88 ± 0.88 ^Ccd^	82.03 ± 0.50 ^CDd^
		Margarine	88.09 ± 0.81 ^Ca^	87.21 ± 0.59 ^CDa^	85.94 ± 1.08 ^Cb^	83.88 ± 0.68 ^Dc^	83.24 ± 0.39 ^Dc^	82.64 ± 0.43 ^Cc^	81.03 ± 0.73 ^Dd^
	−60	Castor oil	90.99 ± 0.54 ^Ba^	91.09 ± 0.97 ^Ba^	90.22 ± 1.07 ^Ba^	88.62 ± 0.99 ^Bb^	88.34 ± 0.63 ^Bbc^	87.94 ± 0.89 ^Bbc^	87.02 ± 0.68 ^Bc^
		Orange oil	95.26 ± 0.76 ^Aa^	95.10 ± 0.71 ^Aa^	94.72 ± 0.83 ^Aa^	93.33 ± 0.46 ^Ab^	92.78 ± 0.29 ^Ab^	92.17 ± 0.65 ^Abc^	91.25 ± 0.53 ^Ac^
		Palm oil	88.39 ± 0.44 ^Ca^	88.02 ± 0.49 ^Ca^	87.33 ± 1.09 ^Ca^	85.81 ± 0.50 ^Cb^	85.21 ± 0.70 ^Cb^	84.91 ± 0.47 ^Cbc^	83.84 ± 0.77 ^Cc^
		Shortening	87.24 ± 0.49 ^Ca^	87.18 ± 0.99 ^Ca^	86.56 ± 0.55 ^Ca^	84.74 ± 0.77 ^Cb^	84.33 ± 0.64 ^Cb^	83.84 ± 0.58 ^CDbc^	82.96 ± 0.76 ^CDc^
		Margarine	88.09 ± 0.81 ^Ca^	87.67 ± 0.87 ^Ca^	86.93 ± 0.89 ^Ca^	85.12 ± 0.66 ^Cb^	84.44 ± 0.28 ^Cbc^	83.61 ± 0.32 ^Dc^	81.95 ± 0.73 ^Dd^
Non-cooking	−18	Castor oil	90.93 ± 0.29 ^Ba^	90.03 ± 0.69 ^Ba^	89.89 ± 0.86 ^Ba^	88.28 ± 0.48 ^Bb^	87.54 ± 0.86 ^Bbc^	86.80 ± 0.36 ^Bcd^	85.92 ± 0.89 ^Bd^
		Orange oil	96.74 ± 0.52 ^Aa^	96.05 ± 0.37 ^Aa^	95.74 ± 0.80 ^Aab^	94.76 ± 0.67 ^Abc^	93.95 ± 0.75 ^Acd^	93.22 ± 0.61 ^Ad^	92.08 ± 0.27 ^Ae^
		Palm oil	89.63 ± 0.72 ^BCa^	88.16 ± 0.45 ^Cb^	87.97 ± 0.67 ^Cb^	86.74 ± 0.74 ^Cc^	86.44 ± 0.90 ^BCcd^	85.53 ± 0.25 ^Cd^	84.01 ± 0.44 ^Ce^
		Shortening	89.04 ± 0.89 ^Ca^	88.36 ± 0.14 ^Ca^	88.04 ± 0.67 ^Ca^	86.65 ± 0.47 ^Cb^	85.92 ± 0.62 ^Cbc^	85.15 ± 0.71 ^Cc^	83.99 ± 0.40 ^Cd^
		Margarine	90.94 ± 0.99 ^Ba^	90.39 ± 0.52 ^Ba^	88.36 ± 0.88 ^Cb^	87.38 ± 0.95 ^BCbc^	86.59 ± 0.54 ^BCc^	85.96 ± 0.99 ^BCcd^	85.05 ± 0.83 ^BCd^
	−60	Castor oil	90.93 ± 0.29 ^Ba^	90.46 ± 0.86 ^Ba^	90.14 ± 0.69 ^Ba^	88.59 ± 0.91 ^Bb^	87.88 ± 0.68 ^Bbc^	87.18 ± 0.84 ^Bcd^	86.05 ± 0.62 ^Bd^
		Orange oil	96.74 ± 0.52 ^Aa^	96.45 ± 0.54 ^Aa^	96.16 ± 0.96 ^Aa^	94.63 ± 0.69 ^Ab^	93.86 ± 0.61 ^Abc^	93.11 ± 0.67 ^Ac^	91.97 ± 0.35 ^Ad^
		Palm oil	89.63 ± 0.72 ^BCa^	89.36 ± 0.24 ^Ca^	88.94 ± 0.81 ^BCa^	87.85 ± 0.54 ^BCb^	86.94 ± 0.85 ^BCbc^	86.17 ± 0.49 ^BCc^	85.04 ± 0.39 ^Cd^
		Shortening	89.04 ± 0.89 ^Ca^	88.84 ± 0.27 ^Ca^	88.38 ± 1.08 ^Ca^	86.99 ± 0.54 ^Cb^	86.27 ± 0.48 ^Cbc^	85.53 ± 0.94 ^Cc^	84.04 ± 0.42 ^Dd^
		Margarine	90.94 ± 0.99 ^Ba^	90.64 ± 0.48 ^Ba^	90.22 ± 0.82 ^Ba^	87.84 ± 0.84 ^BCb^	87.32 ± 0.62 ^BCbc^	86.39 ± 0.76 ^BCc^	85.02 ± 0.65 ^Cd^

^(1)^ ST, storage temperature (°C). ^A–D^ Means within a column with different letters are significantly different (*p* < 0.05). ^a–e^ Means within a row with different letters are significantly different (*p* < 0.05).

**Table 7:** Effects of temperature and storage period on the lightness (L*) and redness (a*) of meat analogs supplemented with different vegetable oils.

	ST ^(1)^	Oil	Storage Period (Months)						
			0	1	2	3	4	5	6
L*	−18	Castor oil	63.75 ± 0.18 ^Ca^	63.94 ± 0.66 ^Ba^	63.48 ± 0.48 ^BCa^	62.40 ± 0.62 ^Cb^	61.71 ± 0.99 ^BCbc^	60.87 ± 0.80 ^BCcd^	60.35 ± 0.94 ^Bd^
		Orange oil	65.91 ± 0.18 ^Aa^	66.24 ± 0.64 ^Aa^	65.90 ± 0.41 ^Aa^	64.65 ± 0.75 ^Ab^	63.98 ± 0.48 ^Abc^	63.28 ± 0.75 ^Acd^	62.96 ± 1.04 ^Ad^
		Palm oil	64.63 ± 0.37 ^Ba^	64.34 ± 0.91 ^Ba^	64.13 ± 0.67 ^Ba^	63.73 ± 0.73 ^Ba^	62.61 ± 0.56 ^Bb^	61.59 ± 0.79 ^Bc^	60.89 ± 0.83 ^Bc^
		Shortening	62.81 ± 0.15 ^Da^	62.71 ± 0.85 ^Ca^	62.37 ± 0.88 ^Da^	62.18 ± 0.42 ^Cab^	61.12 ± 1.13 ^Cbc^	60.40 ± 0.82 ^Ccd^	59.70 ± 1.04 ^Bd^
		Margarine	63.85 ± 0.13 ^Ca^	63.42 ± 1.06 ^BCa^	63.05 ± 0.87 ^CDa^	62.97 ± 0.71 ^BCa^	61.88 ± 0.98 ^BCb^	60.84 ± 0.87 ^BCc^	59.69 ± 0.56 ^Bd^
	−60	Castor oil	63.75 ± 0.18 ^Ca^	63.64 ± 0.37 ^Bab^	63.36 ± 0.40 ^Babc^	62.82 ± 0.89 ^Babc^	62.69 ± 1.03 ^Bbcd^	62.49 ± 0.79 ^Bcd^	61.77 ± 0.66 ^Bd^
		Orange oil	65.91 ± 0.18 ^Aa^	66.03 ± 0.57 ^Aa^	65.74 ± 0.60 ^Aa^	65.08 ± 0.88 ^Aab^	65.17 ± 0.80 ^Aab^	64.49 ± 0.70 ^Abc^	63.75 ± 0.96 ^Ac^
		Palm oil	64.63 ± 0.37 ^Ba^	63.66 ± 1.20 ^Bab^	63.43 ± 1.29 ^Babc^	62.87 ± 0.92 ^Bbcd^	62.79 ± 1.15 ^Bbcd^	62.21 ± 0.74 ^Bcd^	61.65 ± 0.82 ^Bd^
		Shortening	62.81 ± 0.15 ^Da^	62.18 ± 1.25 ^Cab^	61.92 ± 1.11 ^Cab^	61.85 ± 0.55 ^Bab^	61.19 ± 0.77 ^Cbc^	60.84 ± 0.36 ^Cc^	60.24 ± 0.22 ^Cc^
		Margarine	63.85 ± 0.13 ^Ca^	63.36 ± 0.39 ^Bab^	63.14 ± 0.57 ^Bab^	62.63 ± 0.67 ^Bbc^	62.37 ± 0.53 ^Bcd^	61.75 ± 0.76 ^BCde^	61.35 ± 0.52 ^Be^
a*	−18	Castor oil	2.42 ± 0.07 ^Cab^	2.45 ± 0.21 ^BCa^	2.10 ± 0.07 ^ABCbc^	2.20 ± 0.20 ^ABabc^	1.87 ± 0.35 ^ABc^	2.03 ± 0.34 ^Bc^	1.92 ± 0.28 ^Bc^
		Orange oil	1.68 ± 0.21 ^Ea^	2.01 ± 0.11 ^Da^	1.73 ± 0.35 ^Ca^	1.81 ± 0.36 ^Ba^	2.12 ± 0.64 ^Aa^	1.94 ± 0.44 ^Ba^	1.81 ± 0.53 ^Ba^
		Palm oil	2.19 ± 0.18 ^Db^	2.24 ± 0.23 ^CDb^	1.94 ± 0.39 ^BCb^	2.00 ± 0.39 ^ABb^	2.30 ± 0.20 ^Aab^	2.69 ± 0.36 ^Aa^	2.65 ± 0.29 ^Aa^
		Shortening	2.86 ± 0.07 ^Aa^	2.83 ± 0.20 ^Aab^	2.45 ± 0.40 ^Abc^	2.41 ± 0.38 ^Ac^	2.21 ± 0.18 ^Ac^	1.11 ± 0.08 ^Cbc^	2.52 ± 0.25 ^Aabc^
		Margarine	2.64 ± 0.18 ^Ba^	2.61 ± 0.30 ^ABa^	2.26 ± 0.19 ^ABb^	2.17 ± 0.29 ^ABb^	1.59 ± 0.37 ^Bc^	1.18 ± 0.11 ^Cb^	2.80 ± 0.16 ^Aa^
	−60	Castor oil	2.42 ± 0.07 ^Ca^	2.32 ± 0.22 ^Aa^	2.12 ± 0.06 ^ABa^	2.06 ± 0.18 ^Aa^	2.11 ± 0.16 ^Ba^	2.00 ± 0.37 ^Aa^	2.16 ± 0.62 ^ABa^
		Orange oil	1.68 ± 0.21 ^Ebc^	1.71 ± 0.25 ^Bbc^	1.42 ± 0.13 ^Cc^	1.35 ± 0.13 ^Bc^	2.31 ± 0.10 ^Ba^	1.91 ± 0.37 ^Ab^	1.86 ± 0.46 ^Bb^
		Palm oil	2.19 ± 0.18 ^Da^	2.31 ± 0.26 ^Aa^	2.08 ± 0.14 ^Bab^	2.17 ± 0.45 ^Aa^	2.29 ± 0.08 ^Ba^	1.78 ± 0.37 ^Ab^	1.97 ± 0.13 ^Bab^
		Shortening	2.86 ± 0.07 ^Aa^	2.57 ± 0.23 ^Aa^	2.25 ± 0.11 ^Ab^	2.11 ± 0.12 ^Ab^	2.64 ± 0.48 ^Aa^	2.17 ± 0.09 ^Ab^	1.94 ± 0.18 ^Bb^
		Margarine	2.64 ± 0.18 ^Ba^	2.47 ± 0.22 ^Aab^	2.24 ± 0.14 ^ABbc^	2.08 ± 0.13 ^Ac^	2.42 ± 0.15 ^ABab^	2.13 ± 0.20 ^Ac^	2.53 ± 0.31 ^Aa^

^(1)^ ST, storage temperature (°C). ^A–E^ Means within a column with different letters are significantly different (*p* < 0.05). ^a–e^ Means within a row with different letters are significantly different (*p* < 0.05).

**Table 8:** Effects of temperature and storage period on the yellowness (b*) and total color difference (ΔE) of meat analogs supplemented with different vegetable oils.

	ST ^(1)^	Oil	Storage Period (Months)						
			0	1	2	3	4	5	6
b*	−18	Castor oil	16.75 ± 0.20 ^BCa^	16.56 ± 0.21 ^Ca^	16.18 ± 0.28 ^Cab^	15.34 ± 0.45 ^Cc^	15.52 ± 0.51 ^Bbc^	15.07 ± 1.07 ^Bc^	14.87 ± 0.85 ^BCc^
		Orange oil	19.03 ± 0.47 ^Aa^	19.08 ± 0.37 ^Aa^	18.65 ± 0.50 ^Aab^	17.91 ± 0.54 ^Abcd^	18.21 ± 0.90 ^Aabc^	17.65 ± 1.03 ^Acd^	17.19 ± 0.82 ^Ad^
		Palm oil	16.76 ± 0.18 ^BCa^	16.58 ± 0.17 ^Ca^	16.23 ± 0.19 ^Cab^	15.68 ± 0.39 ^BCbc^	15.30 ± 0.97 ^Bcd^	14.82 ± 0.86 ^Bde^	14.25 ± 0.60 ^Ce^
		Shortening	16.60 ± 0.24 ^Ca^	16.60 ± 0.23 ^Ca^	16.40 ± 0.35 ^Cab^	15.70 ± 0.52 ^BCbcd^	15.87 ± 0.49 ^Babc^	15.33 ± 0.90 ^Bcd^	14.97 ± 0.81 ^BCd^
		Margarine	17.09 ± 0.19 ^Ba^	17.11 ± 0.18 ^Ba^	16.99 ± 0.28 ^Ba^	16.11 ± 0.51 ^Bb^	16.37 ± 0.84 ^Bab^	15.95 ± 0.78 ^Bb^	15.53 ± 0.96 ^Bb^
	−60	Castor oil	16.75 ± 0.20 ^BCa^	16.62 ± 0.25 ^Bab^	16.33 ± 0.18 ^Cabc^	15.60 ± 0.43 ^Ccd^	15.85 ± 0.72 ^Bbcd^	15.37 ± 0.97 ^Bd^	15.19 ± 0.73 ^Bd^
		Orange oil	19.03 ± 0.47 ^Aab^	19.09 ± 0.60 ^Aa^	18.78 ± 0.49 ^Aab^	18.19 ± 0.25 ^Aabc^	18.04 ± 0.81 ^Abc^	17.72 ± 1.11 ^Ac^	17.44 ± 0.89 ^Ac^
		Palm oil	16.76 ± 0.18 ^BCa^	16.61 ± 0.29 ^Ba^	16.35 ± 0.08 ^Cab^	15.93 ± 0.45 ^BCab^	15.50 ± 0.88 ^Bbc^	15.02 ± 1.19 ^Bc^	14.62 ± 0.77 ^Bc^
		Shortening	16.60 ± 0.24 ^Ca^	16.59 ± 0.31 ^Ba^	16.38 ± 0.23 ^Ca^	16.10 ± 0.51 ^BCab^	15.77 ± 0.91 ^Bab^	15.19 ± 1.03 ^Bbc^	14.85 ± 0.87 ^Bc^
		Margarine	17.09 ± 0.19 ^Ba^	16.99 ± 0.11 ^Bab^	16.80 ± 0.39 ^Bab^	16.31 ± 0.53 ^Bbc^	15.68 ± 0.77 ^Bcd^	15.06 ± 0.92 ^Bde^	14.72 ± 0.49 ^Be^
ΔE	−18	Castor oil	-	0.62 ± 0.37 ^Cc^	0.86 ± 0.17 ^BCc^	2.04 ± 0.47 ^Bb^	2.48 ± 1.06 ^Ab^	3.55 ± 0.52 ^Aa^	4.06 ± 0.48 ^Aa^
		Orange oil	-	0.79 ± 0.20 ^BCd^	0.73 ± 0.27 ^Cd^	1.73 ± 0.89 ^Bcd^	2.37 ± 0.42 ^Abc^	3.11 ± 0.93 ^ABab^	3.57 ± 1.14 ^Aa^
		Palm oil	-	0.79 ± 0.53 ^BCe^	1.01 ± 0.29 ^BCde^	1.54 ± 0.64 ^Bd^	2.65 ± 0.51 ^Ac^	3.76 ± 0.60 ^Ab^	4.60 ± 0.55 ^Aa^
		Shortening	-	1.59 ± 0.83 ^Ba^	1.51 ± 0.60 ^Ba^	1.59 ± 0.14 ^Ba^	1.43 ± 0.46 ^Ba^	2.49 ± 0.55 ^BCa^	2.34 ± 1.03 ^Ba^
		Margarine	-	3.20 ± 1.06 ^Aa^	2.87 ± 0.84 ^Aab^	2.99 ± 0.69 ^Aa^	2.32 ± 0.71 ^ABab^	2.19 ± 0.33 ^Cb^	1.80 ± 0.84 ^Bb^
	−60	Castor oil	-	0.44 ± 0.24 ^Bd^	0.75 ± 0.12 ^Bcd^	1.64 ± 0.72 ^Bbc^	1.62 ± 0.92 ^Abc^	2.09 ± 0.91 ^Aab^	2.69 ± 0.58 ^ABa^
		Orange oil	-	0.73 ± 0.34 ^Bc^	0.77 ± 0.27 ^Bc^	1.34 ± 0.71 ^Bbc^	1.50 ± 0.95 ^Abc^	2.04 ± 1.18 ^Aab^	2.73 ± 1.28 ^ABa^
		Palm oil	-	1.30 ± 0.82 ^Bc^	1.54 ± 0.87 ^Bc^	2.01 ± 0.96 ^ABbc^	2.27 ± 1.38 ^Aabc^	3.08 ± 1.24 ^Aab^	3.70 ± 1.02 ^Aa^
		Shortening	-	1.24 ± 1.10 ^Ba^	1.22 ± 0.83 ^Ba^	1.27 ± 0.45 ^Ba^	1.37 ± 0.48 ^Aa^	1.73 ± 0.81 ^Aa^	2.23 ± 0.76 ^Ba^
		Margarine	-	3.14 ± 0.38 ^Aa^	2.98 ± 0.53 ^Aa^	2.64 ± 0.63 ^Aa^	2.62 ± 0.74 ^Aa^	2.66 ± 0.99 ^Aa^	2.67 ± 0.51 ^ABa^

^(1)^ ST, storage temperature (°C). ^A–C^ Means within a column with different letters are significantly different (*p* < 0.05). ^a–e^ Means within a row with different letters are significantly different (*p* < 0.05).

**Table 9:** Effects of temperature and storage period on the total cell count and total coliform group bacteria in meat analogs supplemented with different vegetable oils.

	ST ^(1)^	Oil	Storage Period (Months)				
			0	1	2	3	4
Total cell counts (log CFU/g)	−18	Castor oil	ND ^(2)^	ND	3.23	3.58	3.68
		Orange oil	ND	ND	2.56	3.25	3.29
		Palm oil	ND	ND	3.25	3.66	3.70
		Shortening	ND	ND	3.37	3.78	3.84
		Margarine	ND	ND	3.43	3.74	3.84
	−60	Castor oil	ND	ND	2.97	3.53	3.59
		Orange oil	ND	ND	2.43	2.97	3.18
		Palm oil	ND	ND	2.99	3.45	3.56
		Shortening	ND	ND	2.97	3.41	3.60
		Margarine	ND	ND	3.00	3.51	3.64
Total coliform group bacteria (log CFU/g)	−18	Castor oil	ND	ND	ND	ND	ND
		Orange oil	ND	ND	ND	ND	ND
		Palm oil	ND	ND	ND	ND	ND
		Shortening	ND	ND	ND	ND	ND
		Margarine	ND	ND	ND	ND	ND
	−60	Castor oil	ND	ND	ND	ND	ND
		Orange oil	ND	ND	ND	ND	ND
		Palm oil	ND	ND	ND	ND	ND
		Shortening	ND	ND	ND	ND	ND
		Margarine	ND	ND	ND	ND	ND

^(1)^ ST, storage temperature (°C). ^(2)^ ND, not detected.

## Data Availability

Data are contained within the article.
